# Peripheral oxytocin but not vasopressin concentrations are associated with language skills in young autistic children

**DOI:** 10.3389/fpsyt.2026.1854532

**Published:** 2026-08-12

**Authors:** Qin Li, Weihua Zhao, Dan Xu, Jiao Le, Juan Kou, Chunmei Lan, Xinjie Xu, Fanchao Meng, Xi Li, Xiaojing Shou, Rong Zhang, Keith M. Kendrick

**Affiliations:** 1 School of Foreign Languages, Chengdu University of Traditional Chinese Medicine, Chengdu, China; 2 The Clinical Hospital of Chengdu Brain Science Institute, Ministry of Education (MOE) Key Laboratory for NeuroInformation of Ministry of Education, University of Electronic Science and Technology of China, Chengdu, China; 3 China-Cuba Belt and Road Joint Laboratory on Neurotechnology and Brain-Apparatus Communication, University of Electronic Science and Technology of China, Chengdu, China; 4 School of Basic Medical Sciences, Chengdu University of Traditional Chinese Medicine, Chengdu, China; 5 Institute of Brain and Psychological Sciences, Sichuan Normal University, Chengdu, China; 6 Applied Psychology Department, School of Medicine, Southwest University of Science and Technology, Mianyang, China; 7 Laboratory Animal Research Facility, National Infrastructures for Translational Medicine, Institute of Clinical Medicine, Peking Union Medical College Hospital, Chinese Academy of Medical Science and Peking Union Medical College, Beijing, China; 8 The National Clinical Research Center for Mental Disorders and Beijing Key Laboratory of Mental Disorders, Beijing Anding Hospital, Capital Medical University, Beijing, China; 9 Advanced Innovation Center for Human Brain Protection, Capital Medical University, Beijing, China; 10 National Key Laboratory of Cognitive Science and Learning, Beijing Key Laboratory of Brain Imaging and Connectomics, IDG/McGovern Institute for Brain Research, Beijing Normal University, Beijing, China; 11 Neuroscience Research Institute, Department of Neurobiology, School of Basic Medical Sciences, Peking University, Key Laboratory for Neuroscience, Ministry of Education of China, National Health Committee of China, Beijing, China

**Keywords:** autism, language development, oxytocin, social communication, vasopressin

## Abstract

**Background:**

Autistic children often have language development problems, and language ability is a key contributor to social behavior. There is increasing evidence for altered circulating concentrations of the neuropeptides oxytocin and vasopressin in young autistic children and exogenous treatment with either peptide can improve social symptoms. However, to date, the importance of both tonic endogenous concentrations of these two peptides and increased ones following exogenous administration for language development in autistic children has not been established.

**Methods:**

Here we have firstly investigated the associations between blood oxytocin and vasopressin concentrations and vocabulary use and other language/communication measures in a cohort of 148 young autistic children (3–7.5 years) and also in saliva samples from another cohort of 40 autistic children (2–6 years). Furthermore, we examined whether specific improvements in language communication occurred in this latter cohort following 6 weeks of intranasal oxytocin treatment administered every other day followed by positive social interactions previously shown to improve overall social symptoms.

**Results:**

Baseline plasma oxytocin, but not vasopressin, concentrations were significantly associated with both vocabulary development and other language/communication measures after controlling for age and gender in the main cohort of children and additionally in a smaller subset of them even if developmental quotient was controlled for. There was a similar association after controlling for age, gender, and the general adaptability composite score between baseline saliva oxytocin concentrations and language/communication scores in the second independent cohort. In this latter cohort, intranasal oxytocin treatment facilitated language/communication scores across several different scales, and improvement was correlated with post-treatment increases in saliva oxytocin concentrations and significantly indirectly mediated by them.

**Conclusions:**

Overall, this analysis provides support for a potentially important role of oxytocin in language development in autistic children and suggests that it may have therapeutic utility for individuals with language problems.

## 1 Introduction

In recent years, there has been increasing interest in potential therapeutic roles for the hypothalamic neuropeptides oxytocin (OT) and vasopressin (VP) in facilitating social function in a variety of disorders, most notably in autism and also in other disorders such as depression, schizophrenia, and social anxiety (see 1, 2). Reduced concentrations of both OT and VP have frequently, although not always, been reported in these disorders with social behavior problems (3; 4; 5–7), and there is some evidence that they are associated with differences in hypothalamic brain volume (8). By contrast, the genetic neurodevelopmental disorder Prader–Willi syndrome, which is characterized by social behavior as well as eating problems, is associated with increased baseline OT concentrations despite reductions in brain oxytocin neurons and receptors (9). However, it is currently not clearly established what contributes to altered OT concentrations.

Social behavior includes a number of different key features, ranging from the motivation to engage in social interactions, attending to and interpreting specific social and emotional cues, and both verbal and non-verbal communication. Interventions with intranasal OT or VP may potentially influence all of these to improve social symptoms in autism—for example, as measured by various clinical scales (notably the Social Responsiveness Scale and Autism Diagnostic Observation Schedule—see Parker et al. (10) and Le et al. (11)), although the overall findings are significant, they are somewhat variable (see Audunsdottir et al. (12)). There is also some evidence that OT intervention improves social behavior in Prader–Willi syndrome (9). These OT intervention studies tend to focus on the effects of OT on social attention, motivation, and reciprocal social interactions and less on the use of language in social interactions, although one small study in autistic adults has reported some improvement in language (13). A key aspect of social behavior is the development of verbal and non-verbal communication skills and in disorders where these skills develop poorly, or even not at all, the overall impact of which on social behavior is significant (14, 15). There is therefore considerable interest in identifying whether therapeutic interventions which generally facilitate social behavior may do so to some extent by influencing the development of communication and, particularly, language.

Oxytocin has been consistently implicated in vocalizations in terms of song learning and the context of its production in avian species (16, 17). In humans, social vocalizations facilitate the release of OT (18), and there is some evidence that baseline OT concentrations are associated with the development of language fluency (19) and verbal communication (20). One study has also reported associations between OT concentrations and pragmatic language (21), although they used unextracted samples in the OT assays, which may contain substances other than OT and are therefore difficult to interpret (22). Several studies have indicated that intranasal administration of OT can facilitate different aspects of language production or comprehension and verbal memory—for example, intranasal OT increased the second formant production of spoken vowels and enhanced neural activity in sensorimotor cortex and dorsal and ventral speech processing regions during anticipation of speaking (23). Intranasal OT has also been reported to modulate the semantic integration of speech comprehension (24), facilitating the understanding of appeal in verbal communication (25) and reciprocal verbal social communication (26). In a clinical context, OT has been reported to facilitate social cognition, including language, in adult autistic individuals (27) and verbal memory in individuals with schizophrenia (28). It has also been hypothesized that OT may influence language development through its interactions with key identified genetic pathways involved in language, notably FOXP2–CNTNAP2 (29).

For VP, there is also evidence for a facilitatory role in song learning in avian species (30) and vocal behavior in frogs (31), but, to date, no studies have reported any effects of intranasal VP in humans, although given the evolutionary closeness of VP with OT (32) and the fact that both can facilitate social behaviors, there may also be effects of VP on language communication in humans.

In the current exploratory study, we have specifically focused on establishing whether peripheral concentrations of OT or VP are associated with language/communication development in young autistic children, without making comparisons with non-autistic children or other developmental disorders. We firstly used data from a large cohort of 148 young autistic children (21 girls) to establish whether baseline blood OT and VP concentrations were associated with scores on a number of language and verbal communication measures as well as possible sex differences since there is evidence for sex-dependent effects of both OT and VP (see Yao and Kendrick (2)). Additionally, we have analyzed data from subscales of measures taken in recent clinical trials reporting the overall significant effects of 6 weeks of intranasal OT given every other day prior to positive social interactions in improving social symptoms in 41 young autistic children (11). Here we have specifically focused on analyzing associations between both baseline and treatment-induced increases in saliva OT concentrations and scores on subscales of clinical measures focused on language and verbal communication. Overall, we hypothesized that baseline OT and VP concentrations would be associated with language/verbal communication abilities, such that intranasal OT intervention would significantly improve these abilities, and this would be associated with or indirectly mediated by the treatment-dependent increase in OT concentrations.

## 2 Materials and methods

### 2.1 Participants

The first cohort (cohort 1) of 148 autistic children (21 girls) was derived from available data from previous studies investigating the effects of an intervention (33) and structural MRI and social subtype differences between young autistic and non-autistic children (34–36). A number of individuals from these studies had been assessed for vocabulary usage and had baseline measurements of plasma OT and VP concentrations, and only children with baseline peptide concentration measures and specific clinical and behavioral measures of language/verbal communication were included in line with the focus of the current study. All of the autistic children met the diagnostic criteria of Diagnostic and Statistical Manual of Mental Disorders IV-Text Revision (DSM-4 or DSM-5) (37, 38), and diagnosis was confirmed using the Autism Diagnostic Observation Schedule (ADOS) (39, 40). Clinical severity was also assessed in all participants using the Child Autism Rating Scale (CARS—41), which includes subscales for verbal and non-verbal communication. Additionally, a proportion of the children (*n* = 65) had been assessed using the Gesell Development Assessment (42, 43), which has a specific subscale for expressive language use and also provides overall development quotient scores across cognitive, social, and motor domains. All of the autistic children in cohort 1 were recruited through pediatric psychiatric clinics and autism rehabilitation training centers in Beijing. They all had total CARS scores of >22, and while a cutoff of ≥30 is normally used as the criterion for classifying an individual as having autism in the majority of studies, we additionally included children with slightly lower scores to increase the range and include all children on the spectrum (*n* = 18 with CARS scores from 22 to 29). The previous studies had all obtained institutional ethical permission from Peking University and written informed consent from the caregivers of the children. Demographics, clinical scores, baseline OT and VP concentrations, and language/communication scale scores are given in **Table 1**.

**Table 1:** Demographics and clinical and language/communication assessment scores.

Measures	M ± SD	Range
Cohort 1 (*n* = 148)		
Age (years)	4.51 ± 1.05	2.21–7.64
Gender M/F	127/21	
CARS total	35.74 ± 5.62	22–50
ADOS total	15.11 ± 3.65	7–24
OT pg/mL	10.74 ± 5.94	2.0–30.9
VP pg/mL	4.82 ± 3.20	2.0–20.5
L1 speak	159.64 ± 87.81	0–232
L1 comprehend	193.18 ± 60.82	0–232
L2 total	376.50 ± 259.81	0–710
CARS verbal	2.68 ± 0.70	1–4
CARS non-verbal	2.62 ± 0.60	1–4
Gesell language (*n* = 65)	51.03 ± 20.02	14–95
Developmental quotient (*n* = 65)	61.74 ± 16.29	20.4–98.2
Cohort 2 (*n* = 40)		
Age (years)	5.01 ± 1.29	2.9–7.2
Gender M/F	38/3	
ADOS-2 total	16.93 ± 4.15	9–23
SRS-2 total	97.74 ± 22.16	69.5 - 148.5
OT pg/mL^a^ ABAS-2 GCA (scale)	8.11 ± 5.15 40.43 ± 21.78	2.0–22.7 10–81
ABAS-2 verbal comm (scale)	3.65 ± 2.88	0–9
ABAS-2 academic/reading (scale)	3.93 ± 2.94	0–9
SRS-2 social communication	35.17 ± 8.01	21.5–50
SCQ social communication	6.63 ± 2.14	3–12

CARS, Children’s Autism Rating Scale; ADOS, Autism Diagnostic Observation Schedule; OT, oxytocin; VP, vasopressin; L1 speak, vocabulary test 1 speak; L1 comprehend, vocabulary test 1 comprehend; L2 total, total score on vocabulary test 2; SRS-2, social responsiveness scale-2; ABAS-2, Adaptive Behavior Assessment System, second edition; GAC, general adaptive composite; SCQ, social communication questionnaire.

^a^ Saliva samples.

The second cohort of 41 autistic children (cohort 2) was from a randomized, placebo-controlled cross-over, with the clinical trial investigating the effects of intranasal OT or placebo administered every other day for 6 weeks followed by a 30-min period of positive social interaction (11). All of the children were recruited from rehabilitation centers and the Maternal and Children’s Hospital in Chengdu and were diagnosed using DSM-5 and additionally using ADOS-2 (44) and the Social Responsiveness Scale-2 (SRS-2) (45), with ADOS-2 and SRS-2 changes being the primary outcome measures. The study found significant improvements in social symptoms. In the current study, we performed an additional analysis of subscales of two of the original measures taken in the trial, but where only total scores were reported, and their relationship with OT concentrations in saliva. These two scales, the Adaptive Behavior Assessment System, second edition (ABAS-2; 46) and the social communication questionnaire (SCQ; 47) both have language-relevant subscales (ABAS-2: Verbal Communication and Reading/Functional academic subscales; SCQ: Social communication subscale). The ABAS-2 also provides an overall assessment of the level of development across conceptual, social, and practical domains in an overall general adaptability and composite (GAC) score. We also included the SRS-2 social communication subscale for comparison, which was reported in the original study. The original study had ethical permission from the University of Electronic Science and Technology of China ethical committee, had written informed consent obtained from the caregivers of the children, and was pre-registered as a clinical trial (ChiCTR1900023774). The demographic details and relevant test and scale scores are given in **Table 1**.

### 2.2 Language test and clinical measures of communication language ability

In cohort 1 children, language proficiency and skills (performance) in terms of vocabulary use were evaluated using two validated Chinese vocabulary tests intended for non-autistic children and completed by caregivers, one for very young children (12–16 months) with 232 items in 14 categories (test 1) and separate assessments of receptive and expressive language and the other one with 710 items in 20 categories intended for older pre-school children (17–30 months—test 2) which assesses them combined (48). In combination, these two tests can effectively capture the wide range of vocabulary abilities in young autistic children. The Chinese vocabulary tests developed by Hao et al. (48) are an adaptation of the widely used parental report-based MacArthur Bates Communicative Development Inventory (CDI) (49) and have been described in detail in a previous publication (35). These two tests were originally given to 884 Chinese children aged 12–30 months (48), and the results were well matched in terms of speed of development with those of CDI assessments conducted in other languages, with the primary changes occurring on the second test from a median of around 59 words at 17 months (8%) to 594 words at 30 months (84%). The Cronbach’s alpha for the Chinese CDI is >0.9 (50). All autistic children in the current study were also assessed for verbal and non-verbal communication using specific CARS subscales. In a proportion of children (*n* = 65), expressive language proficiency was further assessed using the expressive language subscale of Gesell Developmental Schedule (GDS). The GDS is a standardized instrument used to evaluate a child’s developmental performance across age, including gross motor, fine motor, adaptive, expressive language, and social domains, with good reliability and validity, and is carried out by a qualified clinician. It is designed for children from 2.5 to 9 years and has been validated in a Chinese population with the language subscale achieving a Cronbach’s alpha of 0.95 and a test–retest correlation coefficient of 0.99 (51). Given the focus of this study on language development, we only specifically included analysis of the expressive language subscale of the Gesell, although we also used the developmental quotient from it (average across all subscales) to provide a covariate to control for overall developmental score.

For cohort 2, as mentioned above, language ability was assessed using the two language-relevant subscales of ABAS-2 (the verbal communication and academic/reading subscales). ABAS-2 is a measure of development and adaptability in children (46) similar to Gesell although with some different domains. The social communication subscales of the SCQ and SRS-2 were also used as indicators of language/communication development. ABAS-2, SRS-2, and SCQ questionnaires were completed by the caregivers before and after the placebo and OT arms of the intervention. The GAC score was also used from ABAS-2 as a covariate to help distinguish language development associations from those associated with general developmental adaptability.

### 2.3 Sample collection and assays for OT and VP concentrations

For all subjects included in cohort 1, blood samples were taken by trained nurses, and after centrifugation, the plasma was stored at -80°C until assayed for OT or VP concentrations using commercial ELISAs (ENZO, USA kit ADI-901-153A for OT and ADI-900-017A for VP). For all subjects in cohort 2, saliva samples were collected via a passive drool method prior to the start of the trial and immediately prior to the OT intervention and again 24–48 h after the end of OT treatment to avoid contamination by the final intranasal dose of OT (see 11). Samples were collected onto ice and subsequently stored at -80°C until assayed for OT. Oxytocin concentrations were analyzed in duplicate using the same ELISA assay and protocol as those for cohort 1. For all of the assays, a standard prior extraction step and 4× concentration protocol was performed in accordance with the manufacturers’ recommended protocol. For both OT and VP assays, detection sensitivity was 2 pg/mL, and inter- and intra-assay variations were <10%. The protocols as well as ELISA kits for OT measurements were identical for the two different cohorts. Values below the detection threshold were assigned the minimum detection value.

### 2.4 Statistics

All statistical calculations were performed within SPSS 25.0 or using R, with details of the codes used provided in the **Supplementary Material**. For the study involving cohort 1 associations between the six different language ability scores measured and baseline plasma, OT and VP concentrations were analyzed using Spearman partial correlation controlling for both age and gender. Although relatively few girls (*n* = 21) were included in cohort 1 and the main analysis included gender as a covariate, we nevertheless conducted preliminary analyses using partial correlation analysis separately for male and female to more fully investigate possible sex differences in association with baseline OT and VP concentrations. FDR (*q* = 0.05) correction using the Benjamini–Hochberg adjustment was applied to *p*-values to control for multiple comparisons involving the six measures included, and effect sizes were provided using the coefficient of determination *r*^2^. Differences in correlation strengths between variables were analyzed using Fisher’s *z* for independent samples and using the Cocor package in R (52) for dependent samples.

For the study involving cohort 2, as in the original trial, we analyzed difference scores for effects of OT versus placebo (PLC) interventions on the specific language relevant subscales of ABAS-2, SRS-2, and SCQ using a general linear model, with age, gender, and treatment order as covariates and with *p*-values FDR-corrected and effect sizes calculated using Cohen’s *d*. Spearman partial correlations controlling for potential effects of age and gender were used to analyze associations between relevant subscales of ABAS-2, SRS-2, and SCQ and baseline OT saliva concentrations similar to cohort 1. Additionally, Spearman partial correlation was used to analyze associations between the four measures and post-intervention increases in saliva OT concentration following the 6-week OT intervention. In all cases, FDR (*q* = 0.05) correction was performed for the four measures, and the *r*^2^ value was computed as a measure of effect size. To examine whether the effect of OT treatment on language/communication outcome measures was mediated by post-intervention changes in OT concentrations, we conducted a mediation analysis based on the causal steps approach proposed by Baron and Kenny (53). The analysis was performed in R within a linear mixed modeling (LMM) framework to accommodate the repeated-measures design, and then a bootstrapping (5,000×) step preserving the paired cross-over structure of the data was included to provide 95% confidence intervals and test the significance of indirect effects using *R*. The indirect effect was calculated as a × b and interpreted as statistically significant if the 95% bootstrap confidence interval did not include zero. Age, gender, GAC scores, and treatment order were included as covariates (see **Supplementary Materials** for details).

## 3 Results

### 3.1 Association between baseline oxytocin and vasopressin concentrations and language/communication scores

**Figure 1:**
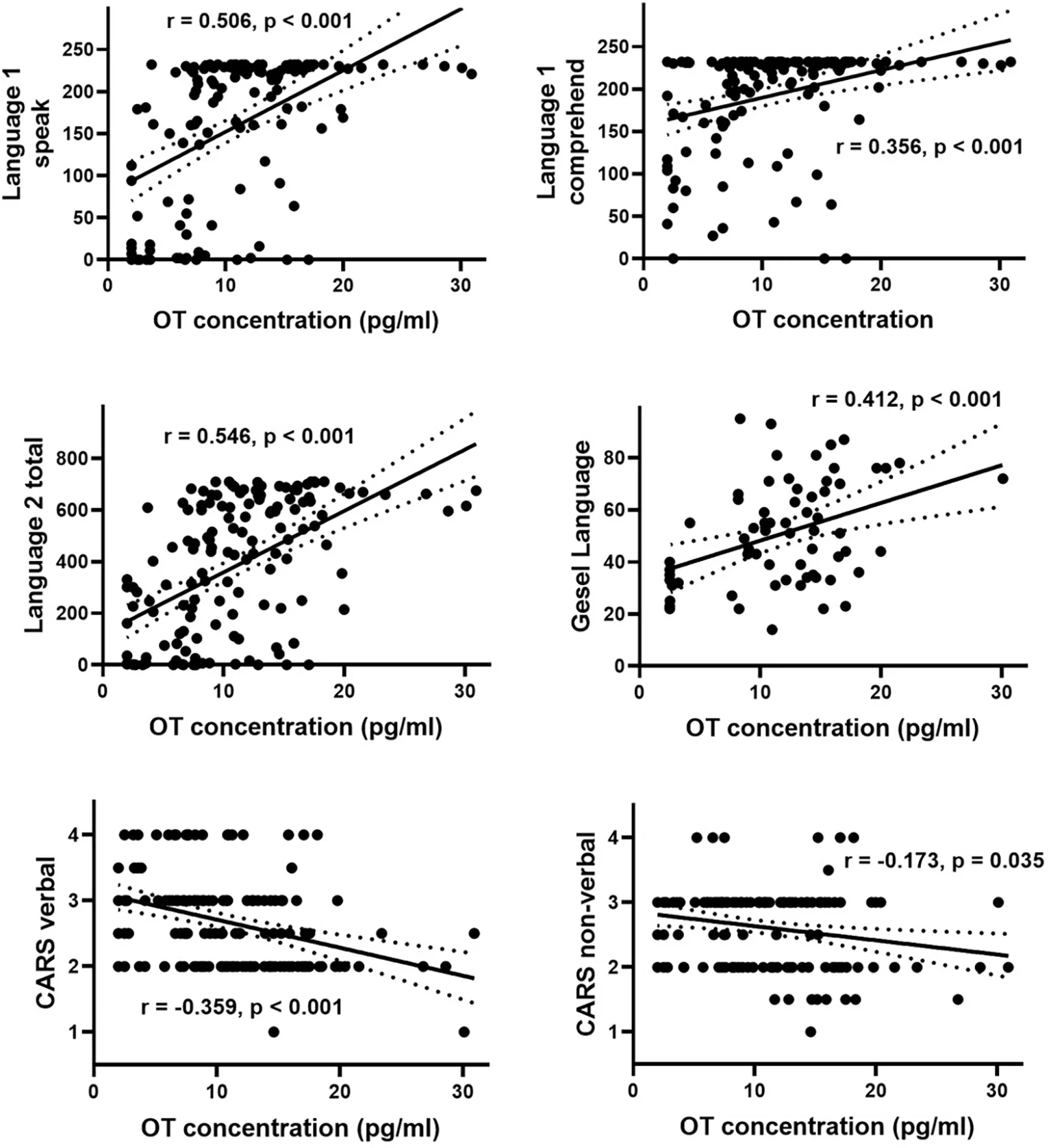
Scatter plots and 95% confidence limits showing correlations between language/communication measures and oxytocin and vasopressin concentrations in cohort 1 subjects. Partial correlation values are given, controlling for age and gender.

**Table 2** shows that in cohort 1 there was a significant partial correlation (controlling for age and gender) between baseline OT concentrations and all six measures of communication/language (vocabulary test 1—expressive (spoken) *r* = 0.506, pFDR < 0.001; receptive (comprehended) *r* = 0.356, pFDR = 0.001; vocabulary test 2 *r* = 0.546, pFDR < 0.001; CARS verbal—*r* = -0.359, pFDR < 0.001, non-verbal—*r* = -0.173, pFDR = 0.035; Gesell expressive language—*r* = 0.412, pFDR < 0.001). The association between OT and expressive language vocabulary (*r* = 0.506, pFDR < 0.001) was significantly stronger than that for receptive language scores (*r* = 0.356, pFDR < 0.001, COCOR: *z* = 3.0473, *p* = 0.0023 two-tailed). For the CARS, there was also a significant correlation with both the verbal communication (*r* = -0.359, pFDR < 0.001) score with non-verbal communication (*r* = -0.173, pFDR = 0.035) subscale scores, with the non-verbal correlation being significantly less (COCOR: *z* = -2.4691, *p* = 0.0135 two-tailed). There were, however, no significant correlations between any of these scales and baseline VP concentrations (all *r*s from – 0.015 to 0.08 and pFDRs from 0.951 to 0.990; see **Table 2**). Scatter plots for associations with OT are shown in **Figure 1**. We conducted a further exploratory analysis on the smaller subset of subjects with Gesell developmental quotient (DQ) scores (*n* = 65), using it as an additional covariate together with age and gender to help assess the extent to which the associations between baseline OT and VP concentrations and the measures of communication/language were influenced by the overall level of cognitive (including language), social, and motor skill development. This analysis revealed smaller partial correlations between the communication/language scores and baseline OT concentrations. Nevertheless, some measures still remained significant after FDR correction despite the smaller cohort of subjects (vocabulary test 2 and Gesell expressive language—see **Supplementary Table 1**). However, while the differences between spoken and comprehended language and OT in vocabulary test 1 remained (*z* = 3.166, *p* = 0.0015), they did not remain between CARS verbal and non-verbal scores (*z* = 0.182, *p* = 0.8556). Correlations with OT were also significantly different from those with VP except for vocabulary test 1 (see **Supplementary Table 1**). As expected, there were strong significant correlations between DQ scores and communication/language scores given that the DQ includes language assessments (ranging between *r* = 0.59 – 0.88, *p <*0.001, Spearman). However, the correlations between DQ scores and OT (*r* = 0.1811, *p* = 0.149) and VP (*r* = -0.1016, *p* = 0.4206) were weak and not significant.

**Table 2:** Spearman partial correlations between oxytocin and vasopressin plasma concentrations and measures of language/communication in male and female autistic children combined in cohort 1 (age- and gender-controlled).

Measures	Partial correlation *r*	*r* ^2^	*t*	pFDR
Oxytocin				
L1 speak	0.506***	0.256	6.687	<0.001
L1 comprehend	0.356***	0.127	4.342	<0.001
L2 total	0.546***	0.298	7.875	<0.001
CARS verbal	-0.359***	0.129	-4.647	<0.001
CARS non-verbal	-0.173*	0.030	-2.123	0.035
Gesell language	0.412***	0.170	3.592	<0.001
Vasopressin				
L1 speak	-0.015	<0.001	-0.169	0.990
L1 comprehend	-0.051	0.003	-0.574	0.951
L2 total	0.001	<0.001	0.012	0.990
CARS verbal	0.080	0.006	0.954	0.951
CARS non-verbal	0.080	0.006	0.961	0.951
Gesell language	0.061	0.004	0.478	0.951

Effect sizes are indicated by *r*^2^.

***p < 0.001; **p < 0.01; *p < 0.05 (two-tailed oxytocin vs. vasopressin correlations).

### 3.2 Sex differences in associations between baseline oxytocin and vasopressin concentrations and language communication scores

There is evidence for sex differences in language development in autism (54); so, although we controlled for gender in the partial correlation initial analysis, we nevertheless also performed a separate exploratory analysis (cohort 1) to assess potential sex differences in associations between OT and VP concentrations and language/communication scores, although there were only data for 21 girls relative to 127 boys. Overall, the analyses in girls showed that only two measures (vocabulary test 1 speak and vocabulary test 2 total) achieved significance after FDR correction compared to those of boys where 5/6 were significant (the exception being the CARS non-verbal scale). Interestingly, there was a significantly stronger association in girls for the non-verbal CARS subscale score compared to boys (partial *r* = -0.562 vs. -0.123, *z* = -2.03, *p* = 0.042). For boys, the difference in associations between spoken and comprehended language vocabulary scores and OT was also significant (*z* = -2.078, *p* = 0.037) (see **Supplementary Table 2**). For VP, on the other hand, the girls showed a trend toward a greater positive correlation between non-verbal CARS scores compared to the boys, but the difference did not achieve significance (*r* = 0.334 vs. 0.029, *z* = 1.26, *p* = 0.332). Similarly, for the Gesell expressive language subscale, girls showed a stronger association with VP concentrations than boys (*r* = 0.383 vs. 0.013), but again this was not significant (*z* = 0.97, *p* = 0.332). Furthermore, while correlations between OT and VP concentrations were significantly positive in boys (*r* = 0.402, *p* < 0.001), they were more negative in girls (*r* = -0.208, *p* = 0.367), and this sex difference was significant (*z* = 2.03, *p* = 0.0424).

### 3.3 Associations with baseline pre-intervention oxytocin concentrations and improvements in language/communication scores in clinical assessments after 6 weeks of intranasal oxytocin administration

**Figure 2:**
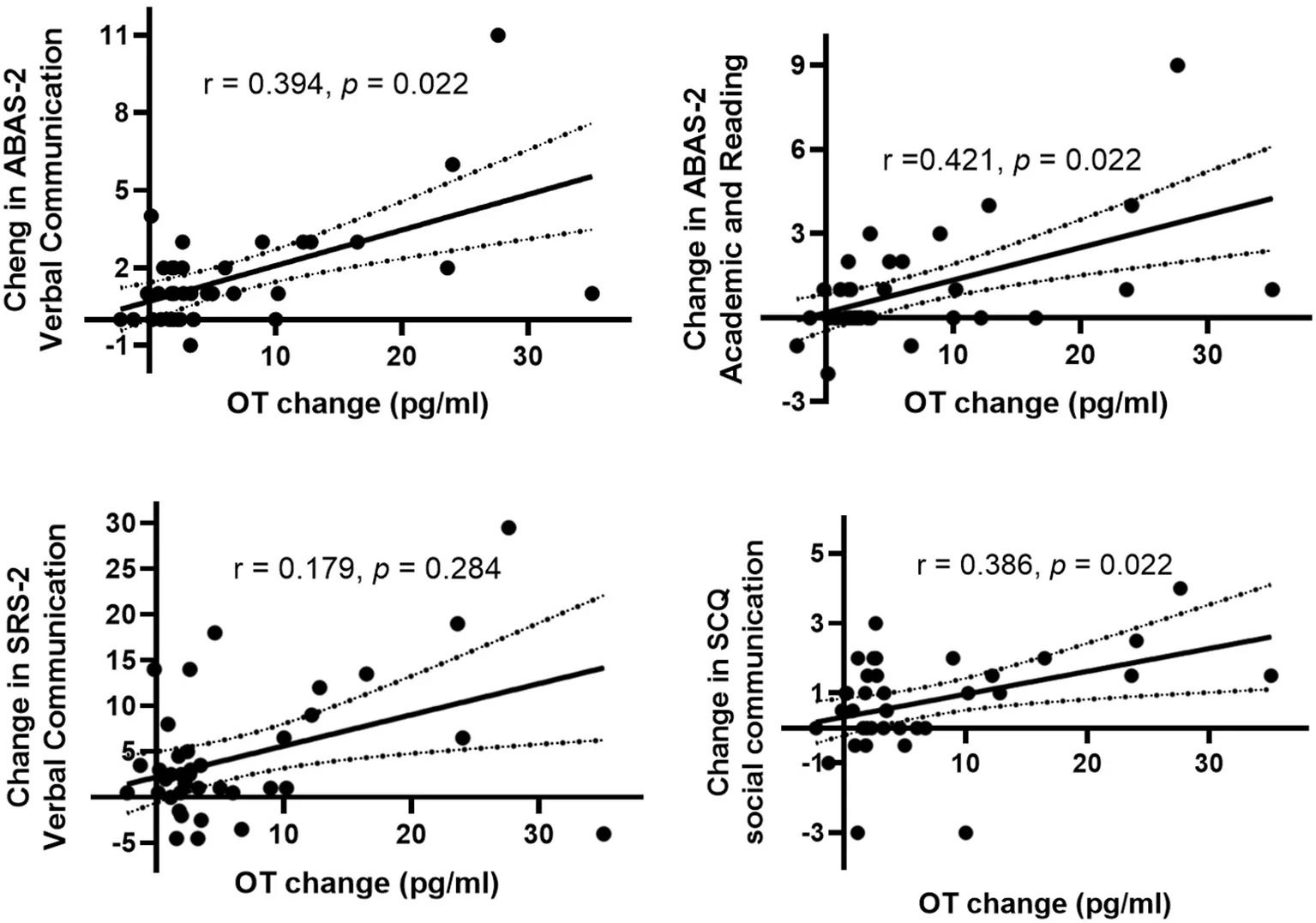
Scatter plots and 95% confidence limits showing correlations between post-intervention increases in saliva oxytocin (OT) concentrations and changes in language/communication measures in cohort 2 subjects. Partial correlation values are given, controlling for age, gender, and general adaptive composite (GAC) score.

A total of 40/41 subjects had pre-intervention baseline saliva concentrations. There was a similar pattern of correlations between these OT concentrations and scores on the four measures of language/communication ability prior to the intranasal OT treatment, but only three were significant and could survive FDR correction (one-tailed tests were used given the hypothesis that the same directions of correlations would occur as already established in cohort 1) and controlling for age, gender, and GAC score (ABAS-2 verbal communication—*r* = 0.465, pFDR = 0.005; ABAS-2 academic and reading—*r* = 0.349, pFDR = 0.027; SRS-2 verbal communication—*r* = -0.314, pFDR = 0.032, and SCQ social communication—r = -0.108, pFDR = 0.253; see **Table 3**). For the 6-week interventions where, in the cross-over design, autistic children received both PLC and OT treatment every other day followed by positive social interaction in a random order, the intranasal OT treatment phase produced a significantly greater improvement in scores for all four measures compared to PLC treatment phase controlling for age, gender, and treatment order (ABAS-2 verbal communication: *F*(1, 39) = 22.356, pFDR = <0.001, Cohen’s *d* = 1.79; ABAS-2 academic and reading: *F*(1, 39) = 9.084, pFDR = 0.009, *d* = 0.76; SRS-2 social communication: *F*(1, 39) = 5.646, pFDR = 0.023, *d* = 0.53; SCQ social communication: *F* (1, 39) = 7.668, pFDR = 0.011, *d* = 0.61). Furthermore, three measures were significantly positively associated with post-OT treatment improvements in language/communication scores and the absolute increase in saliva OT (Spearman partial correlations controlling for age, gender, and GAC scores: ABAS-2 verbal communication—*r* = 0.394, pFDR = 0.022, ABAS-2 academic and reading—*r* = 0.421, pFDR = 0.022, SCQ social communication—*r* = 0.386, pFDR = 0.022; SRS-2 social communication—*r* = 0.179, pFDR = 0.284; see **Figure 2** and **Table 3**).

**Table 3:** Partial correlations between basal and post-intervention absolute change in oxytocin saliva concentrations and measures of language/communication in male and female autistic children in cohort 2 (gender, age, and GAC score are controlled).

Partial correlation		*r* ^2^	*t*	pFDR
Baseline oxytocin				
ABAS-2 verbal communication	0.465#	0.216	3.234	0.005
ABAS-2 academic and reading	0.349#	0.122	2.294	0.027
SRS-2 social communication	-0.314#	0.099	-2.040	0.032
SCQ social communication	-0.108	0.012	-0.671	0.506
Oxytocin change				
ABAS-2 verbal communication	0.394*	0.155	2.570	0.022
ABAS-2 academic and reading	0.421*	0.177	2.787	0.022
SRS-2 social communication	0.179	0.032	1.089	0.284
SCQ social communication	0.386*	0.149	2.510	0.022

Effect sizes are given by *r*^2^.

*p < 0.05, two-tailed; #*p* < 0.05, one-tailed (see **Table 1** for abbreviations).

**Figure 3:**
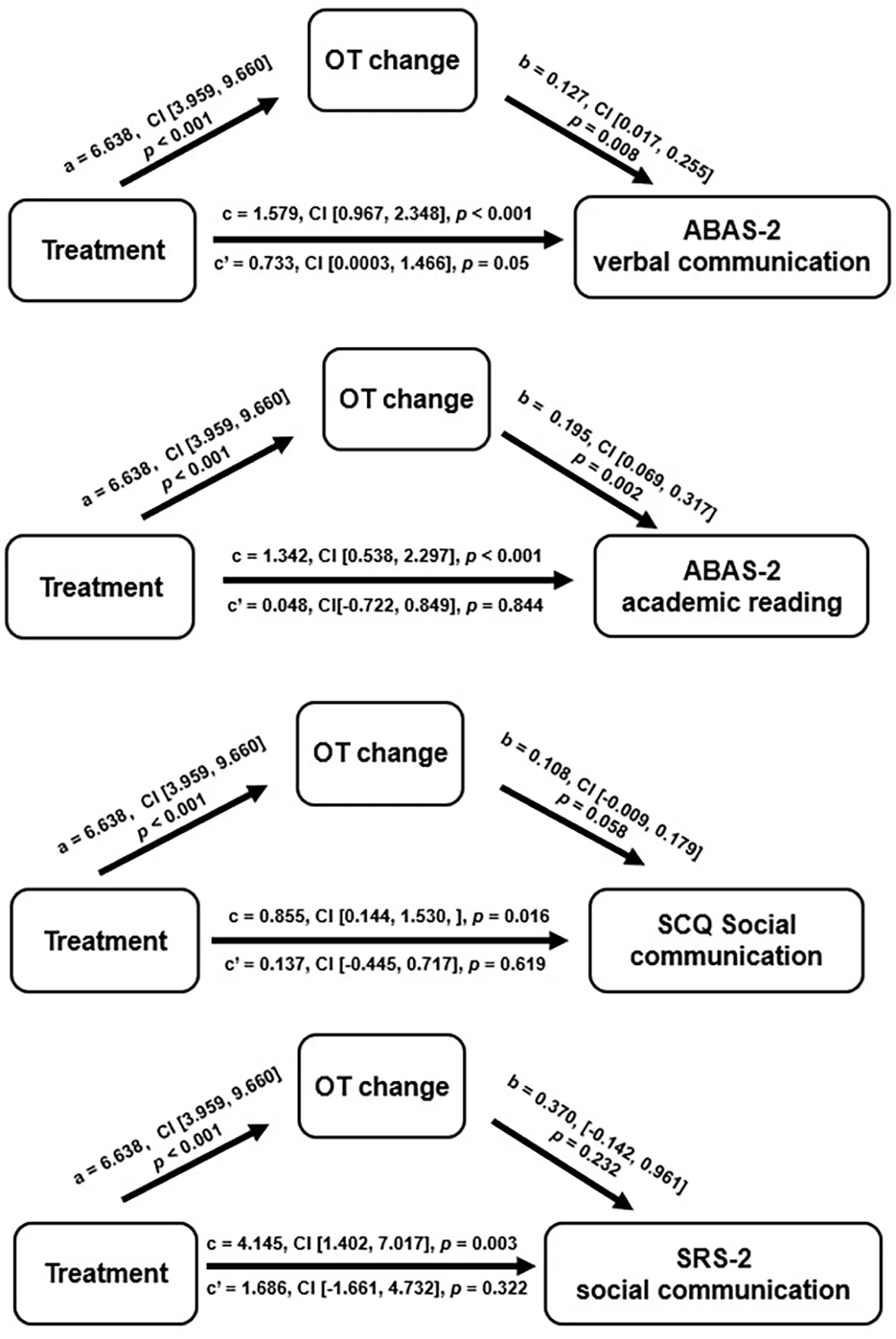
Mediation analysis on all four measures of language/communication showing mediation effects of post-intervention changes in oxytocin (OT) concentrations for placebo vs. OT treatment in cohort 2 subjects. Age, gender, and GAC scores were included as covariates. Bootstrapping (5,000×) was used to calculate 95% CI for all values and showed that ABAS-2 verbal communication had a significant indirect mediation effect of OT changes (i.e., a × b, 95% CI = 0.091–1.888, *p* = 0.0076) as did ABAS-2 academic and reading (95% CI = 0.362–2.436, *p* = 0.002) but not SCQ communication (95% CI = -0.046–1.305, *p* = 0.0504) or SRS-2 communication (95% CI = -0.861–6.69, *p* = 0.232).

An additional mediation analysis controlling for age, gender, and GAC score showed that treatment differences (i.e., PLC vs. OT) within subjects in two measures were significantly indirectly mediated by the change in OT concentrations (ABAS-2—social communication, 95% CI = 0.091–1.888, indirect effect *p* = 0.0076; ABAS academic/reading, 95% CI = 0.362–2.436, indirect effect *p* = 0.002), one marginally significant (SCQ social communication, 95% CI = -0.046–1.305, indirect effect *p* = 0.0584) and one not significant (SRS-2 communication, 95% CI = -0.861–6.69, indirect effect *p* = 0.232—see **Figure 3**). There was a small significant direct effect (i.e., c′) for ABAS-2 social communication (*p* = 0.05) but not for the other measures (see **Figure 3**). This provides some evidence that the effects of OT on language/communication scores were mainly indirectly mediated via post-treatment changes in OT concentrations.

## 4 Discussion

Overall, findings from a new analysis of data from a cohort of 148 young autistic children gathered from previous studies (33; 34; 35; 36) demonstrate a strong association between baseline plasma OT, but not VP concentrations, and a number of language and communication metrics from vocabulary tests and specific subscales of assessments of symptom severity and adaptive behavior measuring language/communication abilities. An exploratory analysis did not identify major sex differences, although while baseline OT and VP concentrations were significantly positively correlated in boys, they tended to be more negatively correlated in girls, although the size of the female cohort was small and findings should be treated with caution. Findings from a new analysis of data from another cohort of 40 young autistic children included in a cross-over clinical trial (11) also revealed a similar pattern for measures of language/communication being associated with baseline OT concentrations. Additionally, in this latter cohort, increases in saliva concentrations of OT following a 6-week intranasal OT intervention followed by positive behavioral interactions were significantly correlated with improvements in language/communication measures and, for the two ABAS-2 measures, significantly indirectly mediated by them. Thus, overall, this study provides initial evidence that OT may particularly help facilitate the development of language and communication skills in young autistic children.

There was consistent evidence from autistic children in the main cohort 1 that baseline plasma OT, but not VP, concentrations were either positively associated with scales measuring language/communication abilities or negatively associated with the severity of problems with them when controlling for age and gender. Effect sizes (*r*^2^) were generally medium to large. Notably, a strong positive association was found in the two tests for vocabulary used in spoken language or comprehension, although with a stronger association of OT concentrations with spoken language. There was also a positive association with expressive language skill using the Gesell language subscale. There was a negative association with the severity of verbal and non-verbal communication problems measured by CARS subscales, although it was significantly stronger for verbal communication. In order to investigate if adaptive development in general rather than language development *per se* was associated with higher baseline OT concentrations, we ran an additional analysis on a small subset of 65 autistic children where DQ scores were available. This revealed a similar pattern of findings, although correlations were reduced and only achieved significance after FDR correction for scores on vocabulary test 2 and Gesell expressive language, although this may have also been contributed to by the small size of the cohort. There was, however, still a significant difference between spoken as opposed to comprehended language in scores from vocabulary test 1, but not between CARS verbal and non-verbal communication, suggesting that initial DQ may have influenced the observed differences between OT concentrations and verbal and no-verbal communication. Indeed DQ was significantly positively correlated with language scores, and language and speech training improves DQ scores (55). Thus, overall, there was some support for OT having a specific association with language/communication which was independent of general adaptive development measured by DQ, although possibly not for verbal vs. non-verbal communication.

There is some evidence for sex differences in language development in autism (54). When boys and girls were analyzed separately, only controlling for age, the boys showed similar significant associations for five of the language/communication measures and baseline OT, but not VP concentrations, although for girls (where the numbers were limited), only a few measures could pass FDR correction. On the other hand, for the CARS non-verbal subscale, while the boys showed a relatively small negative association with OT concentrations (*r* = -0.123), in girls it was significantly larger (*r* = -0.562). This suggests that baseline OT concentrations may be possibly associated with both verbal and non-verbal communication in girls but mainly verbal communication in boys, although this would need to be confirmed in a study including a larger number of girls. For VP, the girls showed a trend toward a positive association between concentrations and CARS non-verbal subscale scores (*r* = 0.334) but not in boys (*r* = 0.013 and 0.029), although this sex difference was not significant. Thus, VP concentrations may actually be possibly associated with more severe non-verbal communication problems in female autistic children, although this again needs further investigation in a larger study. Another observed sex difference was in the association between baseline OT and VP concentrations. In boys, there was a significant positive association (*r* = 0.40), whereas in girls there was a slightly negative association (*r* = -0.271), and this difference was significant. Few studies have reported correlations between baseline OT and VP concentrations, with one using unextracted samples reporting a negative correlation (56) and another, in dogs using extracted samples, a positive correlation (57). However, the general consensus is that extracted and unextracted concentrations of OT in blood are poorly correlated (22), and our findings of significant differences in boys and girls will clearly need to be replicated in a larger study.

Given that both preclinical and clinical trials using intranasal OT and VP as interventions have demonstrated overall similarities in terms of promoting various aspects of social behavior and cognition (see 2; 58), it is perhaps surprising that only OT concentrations showed a close association with language/communication skills. The latter are clearly of great importance for the development of social skills in general (14, 15). Currently, fewer studies have investigated the role of VP in vocal communication in animals (30; 31) and, to date, none in humans. Possibly, the absence of associations with VP concentrations and language/communication scores in blood in the current study may have been contributed by their being somewhat lower than those of OT. On the other hand, there was some evidence to suggest that the higher VP concentrations in girls might be associated with lower communication ability as evidenced by a negative correlation with CARS non-verbal communication symptom scores. Clearly, more studies are required to determine whether VP is influential in this respect or not.

For the smaller cohort of 40 children originally included in the OT intervention clinical trial (11), associations between pre-intervention saliva OT concentrations and measures of language/social communication generally replicated the findings from children in the larger cohort 1, with three out of the four measures (not SCQ social communication) showing the expected direction of correlation (i.e., achieving significant pFDR, one-tailed). In this case, the adaptive development GAC score was included as a covariate together with age and gender, which supports the conclusion that language, rather than other aspects of development, is mainly associated with higher baseline OT concentrations.

In this second cohort of children, the 6-week OT intervention phase followed by positive social interaction in a cross-over clinical trial also produced significant improvements in most of these language/communication measures compared to the same subjects during the PLC intervention phase. While the original trial also reported that OT treatment improved the total SRS-2 and the ABAS GCA scores (11), it did not do so for the total SCQ score. In the current analysis, we found that only the social communication subscale of SCQ showed significant improvement, but not the reciprocal interaction and repetitive and restrictive behavior subscales. One previous study has also reported positive associations between the use of pragmatic language and baseline OT, but not VP concentrations in both male and female autistic children, although using unextracted samples (21) which may measure substances other than OT (22) and not including potentially confounding measures as covariates.

In the second cohort of autistic children, post-OT intervention improvements in three of the measures of language/communication were significantly associated (medium to large effect sizes), with the absolute increase in concentrations of saliva OT observed, the exception being the SRS-2 social communication subscale. Furthermore, a mediation analysis showed that, for the two ABAS-2 measures of language/communication, differences between the PLC and OT treatment phases of the intervention were significantly indirectly mediated by the magnitude of post-treatment changes in OT concentrations. This suggests that the improvements in language/communication seen were dependent to some extent upon how much the intervention increased the OT concentrations, thereby further strengthening the association between OT concentrations and language/communication ability.

At this stage, while there is increasing evidence that OT influences vocal behavior in animals (16, 17), in humans OT concentrations are associated with vocalizations (18), development of language fluency (19), and verbal communication (20). Intranasal administration of OT can facilitate different aspects of language production or comprehension and verbal memory (23–26). Oxytocin treatment has also been reported to facilitate social cognition, including language, in adult autistic individuals (27). However, it is unclear how increases in peripheral OT concentrations might act to facilitate the development of language. Oxytocin administration, either via intranasal or oral routes, may promote the activation of language processing regions via stimulating the vagus (see 2), and indeed vagal stimulation is increasingly demonstrated to improve language learning (59; 60), or possibly by crossing the blood–brain barrier directly into the brain via the olfactory and trigeminal nerves (see Yao and Kendrick (2)). Oxytocin has also been shown to increase the activity and functional connectivity in language processing regions in both non-autistic (61; 23) and autistic (62) populations. Oxytocin is well established as a stimulator of smooth muscle contraction peripherally, and it might affect spoken language by acting on vocal muscles directly, although, to date, there is no evidence for OT receptors in these muscles in humans. More likely is the possibility that OT may influence brain control of vocal musculature, given that it increases activation in sensorimotor cortices and both dorsal and lateral speech processing regions during preparation for speech (23). Oxytocin might also influence language/communication by helping to reduce atypical visual and auditory sensory processing which is prevalent in autistic individuals (63) and can impact adaptive development including language (64). However, a recent clinical trial reporting some improvements in social symptoms in young autistic children after 6 weeks of intranasal OT treatment did not find any effects on sensory processing symptoms (65). Oxytocin has also been reported to influence thermoregulatory (66), metabolic (67), and immune system (68) functioning, all of which could potentially contribute in some way to behavioral symptoms. Additional studies are clearly needed to investigate these different potential mechanisms of action.

The current study has several limitations. Firstly, there were limited detailed assessments of language/verbal communication abilities in the autistic children in both study cohorts and notably in language-specific features. Future studies should include such measures to obtain a clearer understanding of what specific aspect of language development may be particularly influenced by OT and also VP. Secondly, there were only a small number of female autistic children included relative to male children, and this may have obscured some potential sex differences. Thus, our reported sex differences should be treated with caution. Thirdly, we had no data on the IQ of children in the two cohorts studied and only used adaptive development scores using Gesell (DQ) and ABAS-2 (GAC) as covariates to assess for specificity of associations with language/communication relative to more general cognitive development. While these developmental measures are moderately correlated with non-verbal IQ, there can be differences between them in autistic individuals (69–71). However, importantly in the context of the current study, there is some previous evidence that baseline VP and OT concentrations are not correlated with IQ (21). Fourthly, only peripheral neuropeptide concentrations were measured, and they may not accurately reflect those within the brain, although there is some evidence that they do (72). There is also increasing evidence that altered peripheral OT concentrations can mediate functional effects, possibly influencing the brain via peripheral receptors and the vagus (2). Finally, we deliberately restricted our current study to cohorts of autistic children given their well-established heterogeneity in terms of language development and the importance of establishing potential therapeutic interventions. We did not attempt to compare the findings with either non-autistic children or children with other disorders influencing language development, and it will be important in future studies to investigate whether OT is associated more generally with language development.

In summary, we have provided evidence from autistic children using a new analysis of data from two different cohorts of young autistic children from several different previous studies (33; 34; 11; 35; 36; 11) demonstrating a strong association between baseline peripheral OT concentrations and various measures of language/communication skills, particularly in terms of expression. Furthermore, an analysis of additional findings from a recent clinical trial with intranasal OT followed by positive behavioral interaction as an intervention (11) demonstrated that post-treatment increases in saliva OT were also correlated with observed improvements in language/communication skills, and these OT increases significantly indirectly mediated this effect. This supports the possibility that OT may be specifically influential in promoting language/communication development in autistic children as well as their social behavior and cognition more generally, although further studies are required to establish more details of what features of language development may be promoted by OT.

## Data Availability

The raw data supporting the conclusions of this article will be made available by the authors, without undue reservation.
