# Supplementary material for: Peripheral oxytocin but not vasopressin concentrations are associated with language skills in young autistic children
Source: Front Psychiatry. 2026 Aug 12;17:1854532. doi: 10.3389/fpsyt.2026.1854532 (PMC13506699; doi:10.3389/fpsyt.2026.1854532)
Supplement: Supplementary file 1 [file SupplementaryFile1.docx]

Supplementary Material

# 1 Supplementary methods

**Details of statistical approaches and codes in SPSS**

## 1.1 Partial Spearman correlation

To examine the association between OT score and language ability while controlling for age and gender, a partial Spearman correlation was computed using a rank-based residualization approach in SPSS 25.0.

First, rank variables were created for **OT score**, **language ability**, **age**, and **gender (cohort 1 – also an analysis including DQ score in a subset of 65 subjects) and OT score, language ability, age, gender and GAC score (cohort 2)** using the Rank Cases procedure.

Second, to remove the effects of covariates, two linear regressions were performed. In the first regression, the rank of OT score was regressed on the ranks of covariates; in the second regression, the rank of language ability was regressed on the ranks of covariates. Unstandardized residuals were saved from each regression, representing OT score and language ability after adjusting for covariates.

Third, the Pearson correlation coefficient between the two residual variables was calculated. This correlation is equivalent to the Spearman partial correlation between the original variables controlling for covariates, as the residualization procedure accounts for the effects of the covariates on the rank-transformed variables.

Comparisons between computed correlations were analyzed using Fisher’s z for independent samples (i.e. males vs females) and for dependent samples (differences between oxytocin and vasopressin; language test comprehension vs speaking, CARS verbal vs non-verbal) using the Cocor package in R (Diedenhofen and Musch, 2015).

All analyses were conducted using SPSS. Statistical significance was set at α = 0.05.

**Example of the statistical procedure displayed as code-style steps:**

RANK VARIABLES=OT_score Language Age Gender DQ

/RANK

/PRINT=YES

/TIES=MEAN.

REGRESSION

/MISSING LISTWISE

/STATISTICS COEFF OUTS R ANOVA

/CRITERIA=PIN(.05) POUT(.10)

/NOORIGIN

/DEPENDENT ROT

/METHOD=ENTER RAge RGender RDQ

/SAVE RESID.

REGRESSION

/MISSING LISTWISE

/STATISTICS COEFF OUTS R ANOVA

/CRITERIA=PIN(.05) POUT(.10)

/NOORIGIN

/DEPENDENT RLanguage

/METHOD=ENTER RAge RGender RDQ

/SAVE RESID.

CORRELATIONS

/VARIABLES=RES_1 RES_2

/PRINT=TWOTAIL NOSIG

/MISSING=PAIRWISE.

## 1.2 Linear mixed models

Linear mixed models were conducted using SPSS 25.0 to examine the effect of intervention condition on language scores. In the model, **participant ID** was specified as the subject variable for random effects, and **intervention condition** (OT vs. PLC) was specified as the repeated measure, with a **compound symmetry** covariance structure. Fixed effects included **condition, order, age, and gender**, with an additional **condition-by-order interaction** term to assess potential carryover effects. A **random intercept** was included for each participant to account for individual differences. Parameters were estimated using **restricted maximum likelihood**. **Marginal means** were estimated from the model to compare the two conditions, and the significance level was set at α = 0.05.

**Example of the statistical procedure displayed as code-style steps:**

MIXED Language_Score BY Condition Order Gender GAC WITH Age

/FIXED=Condition Order Gender Age GAC Condition*Order | SSTYPE(3)

/METHOD=REML

/PRINT=SOLUTION

/RANDOM=INTERCEPT | SUBJECT(ID)

/REPEATED=Condition | SUBJECT(ID) COVTYPE(CS)

/EMMEANS=TABLES(Condition) COMPARE ADJ(LSD).

MIXED Language_Score BY Condition Order Gender GAC WITH Age

/FIXED=Condition Order Gender GAC Age | SSTYPE(3)

/METHOD=REML

/PRINT=SOLUTION

/RANDOM=INTERCEPT | SUBJECT(ID)

/REPEATED=Condition | SUBJECT(ID) COVTYPE(CS)

/EMMEANS=TABLES(Condition) COMPARE ADJ(LSD).

MIXED Language_Score BY Condition Order Gender GAC WITH Age

/FIXED=Condition Order Gender Age GAC Condition*Order | SSTYPE(3)

/METHOD=REML

/PRINT=SOLUTION TESTCOV

/RANDOM=INTERCEPT | SUBJECT(ID) COVTYPE(VC)

/REPEATED=Condition | SUBJECT(ID) COVTYPE(CS)

/SAVE=RESID

/EMMEANS=TABLES(Condition) COMPARE ADJ(LSD).

## 1.3 Mediation analysis

In the R-based Kenny/Judd within-subject mediation analysis (Baron and Kenny, 1986), the treatment contrast was represented by OT-minus-placebo difference scores. For each participant, the mediator difference was calculated as the OT condition change in OT concentration minus the placebo condition change, and each behavioral outcome difference was calculated in the same direction. The a path was estimated by regressing the mediator difference score on the centered covariates. The ccc path, representing the total treatment effect, was estimated by regressing the outcome difference score on the same covariates. The b and c′ paths were estimated by regressing the outcome difference score on the mediator difference score and covariates. Thus, b represented the association between OT concentration change difference and behavioral outcome difference, whereas c′ represented the direct treatment effect after accounting for the mediator.

All covariates were grand-mean centered before model fitting, including age, gender, treatment order, and GAC, so that the intercept-based treatment effects represented adjusted OT-versus-placebo effects at the average covariate levels. The indirect effect was calculated as a$\times$b. To avoid relying on the normal approximation of the Sobel test, participant-level bootstrap resampling was performed in R to obtain 95% confidence intervals for the indirect effect and for each path coefficient. The bootstrap procedure preserved the paired within-subject structure of the crossover design.

**References:**

Baron RM, Kenny DA. (1986) The moderator–mediator variable distinction in social psychological research: Conceptual, strategic, and statistical considerations. Journal of Personality and Social Psychology, 51(6): 1173.

[Diedenhofen B. Musch J. (2015). cocor: A Comprehensive Solution for the Statistical Comparison of Correlations. PLoS ONE, 10(4): e0121945. doi: 10.1371/journal.pone.0121945](http://journals.plos.org/plosone/article?id=10.1371/journal.pone.0121945)

# 2 Supplementary Figures and Tables

**2.1 Supplementary Tables**

**Table S1**

**Spearman partial correlations between oxytocin and vasopressin plasma concentrations and measures of language ability and communication in male and female autistic children combined from cohort 1 with DQ scores (n = 65; age , gender and DQ controlled as covariates)**

__________________________________________________________________________

**Partial Correlation	r^2^	t	pFDR**

__________________________________________________________________________

**Oxytocin**

L1 Speak	0.222^++^	0.049	1.686	0.116

L1 Comprehend	0.051	0.003	0.382	0.704

L2 Total	0.455*	0.207	4.050	0.001

CARS Verbal	-0.222*	0.049	-1.809	0.113

CARS Non-verbal	-0.236*	0.051	-1.845	0.113

Gesell Language	0.420#	0.176	3.670	0.002

**Vasopressin**

L1 Speak	0.120	0.014	0.887	0.569

L1 Comprehend	0.062	0.004	0.456	0.650

L2 Total	0.212	0.045	1.707	0.236

CARS Verbal	0.071	0.005	-0.578	0.650

CARS Non-verbal	0.198	0.039	1.587	0.236

Gesell Language	0.204	0.041	1.638	0.236

____________________________________________________________________________

* p<0.05 two-tailed # p<0.05 one-tailed for Oxytocin vs vasopressin correlations or ++ p <0.01 for L1 spoken vs L1 comprehended (COCOR)

**Table S2**

**Spearman partial correlations between oxytocin and vasopressin plasma concentrations and measures of language/communication in male and female autistic children in cohort 1 (age controlled)**

|  | **Gender** | **Spearman Partial Correlation** | **r^2^** | **t** | **pFDR** |
| --- | --- | --- | --- | --- | --- |
| Oxytocin |  |  |  |  |  |
| L1 Speak | Male | 0.496***^+^ | 0.246 | 6.051 | <0.001 |
|  | Female | 0.568* | 0.323 | 2.762 | 0.028 |
| L1 Comprehend | Male | 0.341*** | 0.117 | 3.845 | <0.001 |
|  | Female | 0.389 | 0.151 | 1.689 | 0.166 |
| L2 Total | Male | 0.554*** | 0.307 | 7.442 | <0.001 |
|  | Female | 0.567* | 0.321 | 2.998 | 0.024 |
| CARS Verbal | Male | -0.374***^+^ | 0.140 | -4.507 | <0.001 |
|  | Female | -0.279 | 0.078 | -1.266 | 0.221 |
| CARS Non-verbal | Male | -0.123 | 0.015 | -1.391 | 0.167 |
|  | Female | -0.562* | 0.316 | -0.962 | 0.024 |
| Gesell Language | Male | 0.414** | 0.171 | 3.312 | 0.002 |
|  | Female | 0.463 | 0.214 | 1.477 | 0.214 |
| Vasopressin |  |  |  |  |  |
| L1 Speak | Male | -0.010 | <0.001 | -0.109 | 0.971 |
|  | Female | -0.040 | 0.002 | -0.159 | 0.875 |
| L1 Comprehend | Male | -0.080 | 0.006 | -0.843 | 0.971 |
|  | Female | 0.097 | 0.009 | 0.392 | 0.875 |
| L2 Total | Male | -0.003 | <0.001 | -0.036 | 0.971 |
|  | Female | 0.050 | 0.003 | 0.220 | 0.875 |
| CARS Verbal | Male | 0.051 | 0.003 | 0.568 | 0.971 |
|  | Female | 0.227 | 0.051 | 1.015 | 0.646 |
| CARS Non-verbal | Male | 0.029 | 0.001 | 0.321 | 0.971 |
|  | Female | 0.334 | 0.111 | 1.543 | 0.646 |
| Gesell Language | Male | 0.013 | <0.001 | 0.096 | 0.971 |
|  | Female | 0.383 | 0.147 | 1.174 | 0.646 |

CARS = Children’s Autism Rating Scale; ADOS = Autism Diagnostic Observation Schedule; L1 Speak = Vocabulary Test 1 Speak; L1 Comprehend = Vocabulary Test 1 Comprehend; L2 Total = Total score on vocabulary test.

*** p <0.001, ** p < 0.01, * p<0.05 two-tailed, ^+^ p < 0.05 L1 Speak vs L1 comprehension or CARS verbal vs non-verbal; # p<0.05 male vs female
